# CRFK and Primary Macrophages Transcriptomes in Response to Feline Coronavirus Infection Differ Significantly

**DOI:** 10.3389/fgene.2020.584744

**Published:** 2020-12-03

**Authors:** Yvonne Drechsler, Elton J. R. Vasconcelos, Lisa M. Griggs, Pedro P. P. V. Diniz

**Affiliations:** ^1^College of Veterinary Medicine, Western University of Health Sciences, Pomona, CA, United States; ^2^Leeds Omics, University of Leeds, Leeds, United Kingdom

**Keywords:** CRFK, macrophages, feline coronavirus, host response, transcriptome

## Abstract

Coronaviruses are highly infectious and common in many species, including in humans, and agricultural and domestic animals. Host responses play an important role in viral entry, replication, assembly, and pathogenesis, although much is still to be understood, particularly host–virus interactions. Feline coronavirus is highly contagious, and ubiquitous in virtually all cat populations. Host-pathogen interactions have not been studied extensively due to the complex pathogenesis and development of clinical disease. Few studies have investigated cellular host responses to feline coronavirus infection, particularly at early time points. Transcriptome studies based on next-generation sequencing have the potential to elucidate the early responses of cells after viral infection and, consequently, give further insight into the pathogenesis of viruses. The current study aims to characterize and compare the viral- and immune-related differentially expressed genes in response to the coronavirus FIPV across different time points in a cell line which is permissive for productive replication versus primary cells implicated in pathogenesis. When comparing host responses in Crandell-Rees Feline Kidney (CRFK) cells to primary macrophages, many differences were observed with regards to expressed genes and their enrichments for both KEGG pathways and GO terms. CRFK cells which are permissive for productive replication of feline infectious peritonitis virus, showed induction of a large network of immunological and virally induced pathways. In contrast, Macrophages did not show similar host responses, with stronger pathway enrichment in downregulated transcripts. This study provides insights to better understand gene transcription in immune cells compared to epithelial cells discerning pathways relevant to pathogenesis in the early stages of infection.

## Introduction

Coronaviruses are highly infectious and common in many species, including in humans, and agricultural and domestic animals. A significant amount of research has been done on human coronaviruses, due to relatively recent outbreaks of zoonotic viruses such as SARS, MERS and the current SARS-CoV2 (etiologic agent of COVID-19) (Peiris et al., 2003; Hayden et al., 2014; Menachery et al., 2015; Wan et al., 2020). Due to high economic losses, coronaviruses of agricultural animals have also been a focus of research. Coronaviruses infect diverse cell types, such as epithelial and various immune cells (Rossen et al., 1995; Totura et al., 2015). Consequently, host innate immune responses have become of particular interest due to the lack of effective vaccines and the strong innate immune component in pathogenesis (Frieman et al., 2008; Totura and Baric, 2012; Shi et al., 2014).

Host responses play an important role in viral entry, replication, assembly, and pathogenesis, although much is still to be understood, particularly host-virus interactions. Human coronaviruses have been shown to target apoptotic pathways, with virally induced apoptosis occurring in immune cells and target tissues (Collins, 2002; Krahling et al., 2009; Chu et al., 2016). Viral proteins, such as SARS-CoV S, N, E, M, ORF-6, 7a and 9b, activate pro-apoptotic pathways (Lim et al., 2016). Other interactions of the virus with the immune system involve immune evasion of the virus and preventing a robust innate immune response. Several members of the coronavirus family have been shown to inhibit innate immune responses by targeting IFN type I (Spiegel et al., 2005; Kint et al., 2016). Other pathways, including endoplasmic reticulum (ER) stress responses, autophagy, MAPK, and NFκB are modulated by coronaviruses (Fung et al., 2014; Shi et al., 2014; Lim et al., 2016), and these modulations enable the virus to efficiently replicate. Insight into these modulations will further elucidate the pathogenesis of these viruses.

Feline coronavirus (FCoV) is highly contagious, and ubiquitous in virtually all cat populations (Pedersen, 1976; Addie and Jarrett, 1992; Cave et al., 2004; Drechsler et al., 2011). Host-pathogen interactions have not been studied extensively due to the complex pathogenesis and development of clinical disease. Two feline coronaviruses feline enteric coronavirus (FECV) and feline infectious peritonitis virus (FIPV) exist which manifest with different pathologies. The enteric virus, FECV, commonly causes an asymptomatic infection presenting with mild gastrointestinal signs, and can persist in certain individuals (Pedersen et al., 1981). When the infection turns pathogenic, a highly fatal systemic immune-mediated disease develops, so-called feline infectious peritonitis (FIP) (Pedersen et al., 1981). The pathogenesis of FIPV is still not well understood, specifically the early events leading from viral infection to systemic distribution and development of disease.

Feline infectious peritonitis virus infection is thought to be mediated by macrophages distributing the virus systemically, followed by depletion of CD4^+^ and CD8^+^ T-lymphocytes. The exact nature of this process, however, is unknown, but most likely the apoptosis is mediated by signaling cytokines from other cells (Haagmans et al., 1996). The absence of a robust cell-mediated immunity is likely responsible for the fatal outcome of the viral infection. In cats with confirmed FIP, serum tests showed increased inflammatory cytokine expression, and clinical signs are closely correlated with inflammation (Pedersen, 1976, 2019; Dean et al., 2003; Kipar et al., 2006).

Few studies have investigated cellular host responses to feline coronavirus infection, particularly at early time points. Transcriptome studies based on next-generation sequencing have the potential to elucidate the early responses of cells after viral infection and, consequently, give further insight into the pathogenesis of viruses (Radford et al., 2012). Since both FIPV and FECV pathotypes replicate similarly in Crandell-Rees Feline Kidney (CRFK) cells, analyzing the transcriptome of these cells can provide information on replication of the virus and host-pathogen interactions. Recent studies have investigated host responses to FIPV via transcriptome studies in CRFK cells after infection (Harun et al., 2013; Mehrbod et al., 2015). In one of these studies, the gene expression of peripheral blood mononuclear cells from infected cats (Harun et al., 2013) was also investigated. However, due to the important role of immune cells in the disease, analyzing the responses of macrophages is critical to better understanding innate immune responses to feline coronavirus.

The current study aims to characterize and compare the viral- and immune-related differentially expressed genes in response to the coronavirus FIPV across different time points in a cell line which is permissive for productive replication versus primary cells implicated in pathogenesis.

## Materials and Methods

### Cell Culture

CRFK cells (ATCC, Manassas, VA, United States, cat#: CCL-94) were grown as monolayers in Dulbecco’s Modified Essential Medium (DMEM) containing 10% Fetal Bovine Serum (FBS) and 1% Penicillin/Streptomycin at 37°C and 5% CO2.

### Animal Studies

All animal procedures were conducted and approved under the guidelines of the Institutional Animal Care University Committee (IACUC) of Western University of Health Sciences, protocol approval number R10/IACUC/017. Peripheral blood for transcriptome studies was taken from six healthy male, specific pathogen-free (SPF) cats residing in an existing colony at the University of California, Davis. 30–40 ml of blood, equivalent to 1% of body weight or less was collected in heparinized tubes. The ages of cats at the time of blood draw were 5 months up to 2 years (five cats), and 4 years (one cat).

### Monocyte Isolation

Monocytes from peripheral blood were isolated as previously described for canine monocytes (Goto-Koshino et al., 2011) with some modifications. Briefly, the gradient centrifugation steps occurred at 450 × *g* without break at deceleration, and the subsequent washes to remove platelets were performed at 200 × *g* for a total of three washes. Peripheral blood mononuclear cells (PBMCs) were counted, resuspended in RPMI 1640 containing 10% of FBS, 1% of penicillin and streptomycin, and 1 x of non-essential amino acids, and plated at 5 × 10^6^ in 6-well plates. Non-adherent cells were removed after 24 h by vigorously washing with culture medium before infecting cells the following day.

### Viral Infection for Transcriptome

For host transcriptome studies, macrophages were infected with FIPV 79-1146 (ATCC VR2128). The viruses were incubated at a multiplicity of infection (MOI) of 2 in a serum-free OptiMEM (Gibco, Thermo Fisher Scientific, Waltham, MA, United States) for 1 h for virus attachment, washed with OptiMEM, and incubated with fresh supplemented RPMI1640 for an additional 2 or 17 h. Technical replicates for the control, 2 and 17 h for macrophages from each cat were plated and incubated with PBS or the virus, respectively. CRFK cells (including technical replicates) were also infected as the control at an MOI of 1 in OptiMEM, followed by incubation in a supplemented DMEM. Uninfected controls underwent the same process with PBS without the virus. After incubation, the cell culture medium was completely removed and 600 μL of TRIzol (Invitrogen, ThermoFisher Scientific, Waltham, MA, United States) was added to each well, followed by RNA extraction with the ZymoResearch RNA kit (ZymoResearch, Irvine, CA, United States) according to the manufacturer’s instructions. The RNA quality was evaluated via the Bioanalyzer (Agilent, Santa Clara, CA, United States) and sent (1 μg per sample) for mRNA sequencing to Novogene, Inc. (Sacramento, CA, United States).

### Quality Control of RNA Sequence Data

Quality control on seven CRFK (two uninfected controls, two 2 h post-infection, and three 17 h post-infection samples) and sixteen Macrophage (four uninfected controls, seven 2 h post-infection, and five 17 h post-infection samples) RNA paired-end sequencing libraries was assessed through FastQC^1^. An average of 35 million paired reads were sequenced per sample. Both adapters and low-quality bases (QV < 20) were trimmed from the reads’ extremities with Trimmomatic (Bolger et al., 2014).

### Alignment Against Reference Transcriptomes

Kallisto (Bray et al., 2016) was the algorithm of choice for performing the alignment of all paired reads against the whole *Felis catus* reference transcriptome (*F. catus* NCBI-RefSeq-9.0). An average of 87.5% of the total reads from each sample was mapped onto the cat’s annotated transcriptome. Alternatively, we also attempted to retrieve viral reads for both macrophages and CRFK from the sequenced libraries using Kallisto to align reads against the 11 protein-coding genes from the feline coronavirus (FCoV, NCBI accession number KX722530.1) to verify viral presence in the cells.

### Differential Expression

Read counts tables generated by Kallisto were used as input for differential expression (DE) analyses with EdgeR version 3.8 (Robinson et al., 2010). The generalized linear model (GLM) fitting method was applied followed by a one-way ANOVA-like test (2 h-vs.-Ctrl and 17 h-vs.-Ctrl) with a 0.01 FDR threshold. Datasets were submitted to a multidimensional scaling (MDS) analysis, with the *plotMDS* function from EdgeR, to identify distinct samples clustered in a two dimensions-reduction landscape prior to the start of DE analyses (Drechsler et al., 2020) (Supplementary Figure S1). Additionally, Kallisto-generated transcripts per million (TPM) normalized expression data for each sample was used to plot heatmaps for both cell types using the *heatmap.2* function from the gplots R package, with the embedded Hierarchical Clustering assessment turned on for both rows (transcripts) and columns (samples). All tools described above for differential expression were run within the R environment version 3.5.2.

### Gene Enrichment Analyses Using Both Gene Ontology (GO) Terms and KEGG Pathways

After generating a list of differentially expressed genes (DEGs), we used ClueGO (Bindea et al., 2009) under the Cytoscape version 3.7.1 (Shannon et al., 2003; Smoot et al., 2011) for a gene enrichment analysis relying on the *Felis catus* annotation from both Gene Ontology^2^ and KEGG^3^ consortia. Both enrichment analyses adopted the Hypergeometric test along with the Benjamini and Hochberg *p*-value adjustment method. A 0.05 threshold was set for the latter. For network constructions, we relied on the default *kappa* score parameter for drawing term-term interaction edges. GO enrichment bubble charts were drawn through the “enrichment_chart” function from the “pathfindR” R package (Ulgen et al., 2019).

## Results

### CRFK Transcriptome Shows Robust Gene Expression at 2 h in Response to Feline Coronavirus

Transcriptome analysis of CRFK cells showed robust gene expression at 2 h after infection, with over 1000 genes significantly downregulated, and over 700 genes significantly upregulated more than twofold (Supplementary Table S1 and Supplementary Figure S2A). Despite the large number of genes, KEGG pathways-based gene enrichment analysis at this early time point after infection showed only two enriched cellular processes that were downregulated: axon guidance and choline metabolism in cancer (Supplementary Table S2).

In contrast, there were 14 KEGG pathways enriched for upregulated genes at 2 h after infection with FIPV. Those were associated mostly with immune signaling categories, such as “MAPK signaling,” “chemokine signaling,” “Toll-like receptor signaling,” “T cell receptor signaling,” “TNF signaling,” “RIG-1 like receptor signaling” and viral signaling pathways including those associated with Hepatitis B and Influenza A (Supplementary Table S2).

**FIGURE 1:**
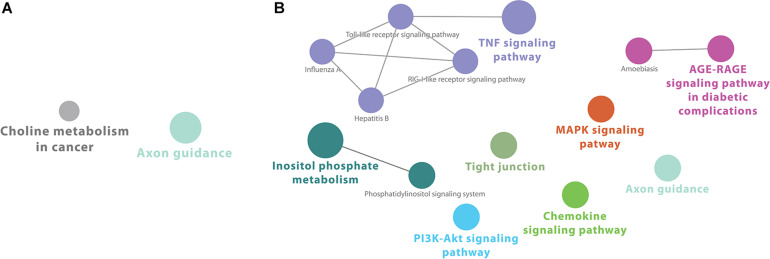
Cytoscape network analysis of **(A)** downregulated and **(B)** upregulated KEGG pathways at 2 h after infection (adj. *p*-value < 0.05). **(A)** Pathways for downregulated genes are not connected. **(B)** For upregulated genes, immunological pathways, particularly involved in response to viruses, form a network that includes “Toll-like receptor signaling,” “RIG-I-like receptor signaling,” “TNF signaling,” and other virally induced pathways. A multi-color node means that genes within that labeled category are also placed/present in other associated higher hierarchy terms, represented by their respective colors elsewhere in the network.

Cytoscape ClueGO-generated network from KEGG-based enrichment shows the interaction of the various pathways at 2 h after infection (Figure 1). Pathways for downregulated genes are not connected (Figure 1A). For upregulated genes, immunological pathways, particularly involved in response to viruses, form a network that includes “Toll-like receptor signaling,” “RIG-I-like receptor signaling,” “TNF signaling,” and other virally induced pathways (Figure 1B).

**FIGURE 2:**
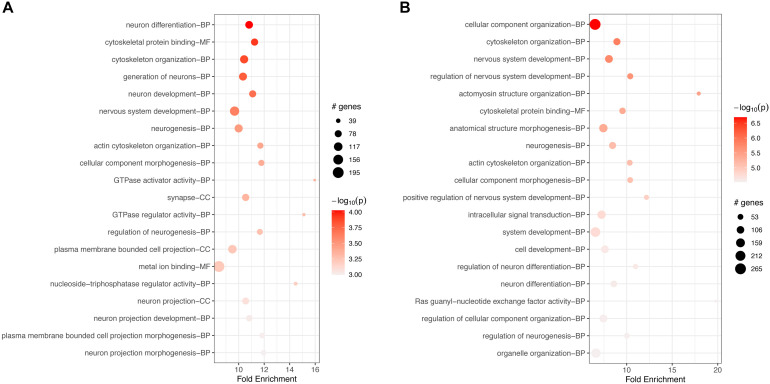
Top 20 most significant GO terms for **(A)** downregulated genes or **(B)** upregulated genes in FCoV-infected CRFK cells 2 h post-infection (adj. *p*-value < 0.05). The top 20 significant terms include neuronal development and differentiation, cellular organization, and in the case of upregulated genes **(B)** also intracellular signal transduction.

GO-based enrichment analyses for v6c5 both downregulated (Supplementary Table S3) and upregulated (Supplementary Table S4) genes showed a large number of biological processes (BP), molecular functions (MF), and cellular components (CC) terms. The top 20 terms for each category are shown in Figure 2. In both cases, down- and upregulated genes, the top 20 significant terms include neuronal development and differentiation, cellular organization, and in the case of upregulated genes also intracellular signal transduction (Figure 2).

### CRFK Transcriptome Shows Strong Immune-Related Gene Expression at 17 h in Response to Feline Coronavirus

KEGG-based enrichment analysis at 17 h after infection shows four enriched pathways for downregulated genes, “MAPK signaling,” “Inositol phosphate metabolism,” “Phosphatidylinositol signaling system,” and “axon guidance,” with axon guidance also downregulated at 2 h (Supplementary Table S5).

For upregulated genes, KEGG-based enrichment showed a robust number of significant pathways at 17 h post-infection, strongly skewing to upregulation of immunological pathways. These included, but were not limited to, several viral pathways (Kaposi, cytomegalovirus, HIV, Epstein-Barr, Hepatitis, Measles, Influenza, and others), “viral protein interaction with cytokine and cytokine receptor,” “Toll-like receptor signaling,” “NF-kappa B signaling,” “JAK-STAT signaling” and many more that play a role in host responses to viral infections (Supplementary Table S5). Overall, 32 pathways were enriched for upregulated genes at 17 h post-infection, with the large majority of these having immunological significance, particularly in the host response to viral infection.

**FIGURE 3:**
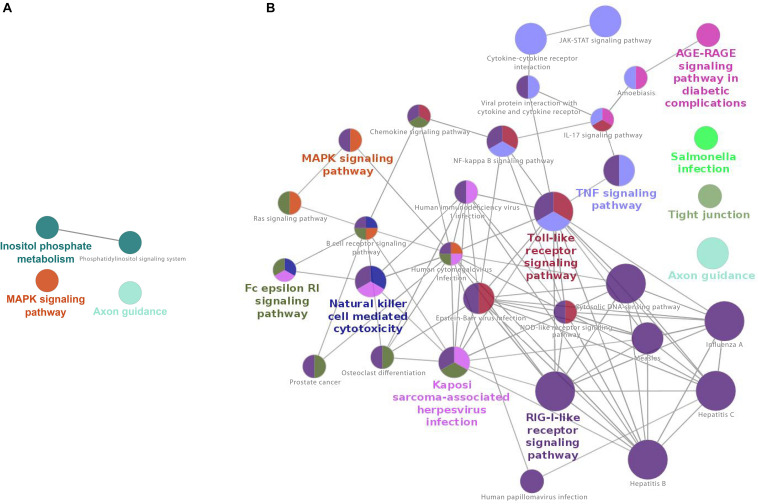
Cytoscape network analysis of **(A)** downregulated and **(B)** upregulated KEGG pathways at 17 h after infection (adj. *p*-value < 0.05) of CRFK. **(A)** Cytoscape network analysis did not show significant interaction of nodes among downregulated pathways, **(B)** The network shows strong interactions between many immunological pathways, specifically those involved in host–virus signaling and interaction. Major nodes are Toll-like receptor signaling, RIG-I-like receptor signaling pathways, and several other virally induced pathways. A multi-color node means that genes within that labeled category are also placed/present in other associated higher hierarchy terms, represented by their respective colors elsewhere in the network.

Cytoscape network analysis did not show significant interaction of nodes among downregulated pathways (Figure 3A). Network analysis of upregulated pathways at this time point shows strong interactions of several immunological and viral pathways, such as “Toll-like receptor signaling,” “RIG-I receptor signaling,” and many other immunological/viral pathways (Figure 3B).

**FIGURE 4:**
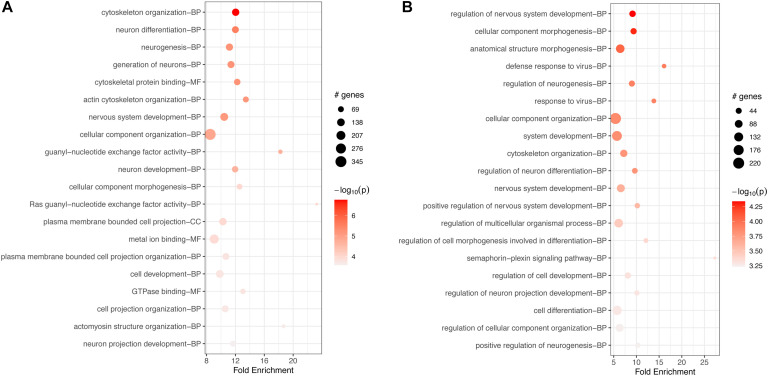
Top 20 most significant GO terms for **(A)** downregulated and **(B)** upregulated genes in FCoV-infected CRFK cells 17 h post-infection (adj. *p*-value < 0.05). **(A)** The top 20 terms for downregulated genes were very similar to the top terms at 2 h, with many processes related to neuronal and cellular development, organization, and differentiation. **(B)** For upregulated genes, the top 20 terms were similar to enrichment terms at 2 h in regards to neuronal and cellular responses. Additional terms found were “response to the virus,” “defense response to the virus,” and the “semaphoring-plexin signaling pathway.”

GO-based enrichment for downregulated genes at 17 h included many of the same terms as enrichment at 2 h (Supplementary Table S6). The top 20 terms were also very similar to the top terms at 2 h, with many processes related to neuronal and cellular development, organization, and differentiation (Figure 4A).

GO-based analysis for upregulated genes also showed a large number of enriched terms (Supplementary Table S7). The top 20 terms, while similar to enrichment terms at 2 h in regards to neuronal and cellular responses, additionally included the terms “response to the virus,” “defense response to the virus,” and the “semaphoring-plexin signaling pathway” (Figure 4B).

### Top 10 Genes Differentially Expressed at 2 h and 17 h in CRFK Cells

A large number of genes were differentially down- or upregulated both at 2 and 17 h. Among the top 10 genes downregulated at both time points, three were the same, heterogeneous nuclear ribonucleoprotein K (transcript variants 1 and 8), and chromosome A3 C20orf194 homolog. Talin 1 was the most downregulated gene at 17 h, while not in the top ten downregulated genes at 2 h (Table 1).

**TABLE 1:** Top 10 genes downregulated at 2 and 17 h in FCoV infected CRFK cells.

Top 10 Genes Downregulated at 2 h			Top 10 Genes Downregulated at 17 h		
	
*RefSeqID*	*Log fold*	*Description*	*RefSeqID*	*Log fold*	*Description*
XM_011288260.3	−13.47	Heterogeneous nuclear ribonucleoprotein K, transcript variant X1*	XM_019816171.1	−14.28	Talin 1, transcript variant X5
XM_023259529.1	−12.75	NCK associated protein 1, transcript variant X1	XM_011288260.3	−13.45	Heterogeneous nuclear ribonucleoprotein K, transcript variant X1*
XM_011283119.3	−12.44	Chromodomain helicase DNA binding protein 8, transcript variant X1	XM_011288264.3	−11.74	Heterogeneous nuclear ribonucleoprotein K, transcript variant X8*
XM_019826358.2	−12.33	Activity dependent neuroprotector homeobox, transcript variant X5	XM_006934701.3	−11.63	Intracisternal A particle-promoted polypeptide, transcript variant X1
XM_003989031.5	−12.09	Carboxypeptidase M, transcript variant X3	XM_023245386.1	−11.52	Dynamin 2, transcript variant X6
XM_019826406.2	−11.99	Zinc finger MYND-type containing 8, transcript variant X10	XM_003989056.4	−11.36	Nucleosome assembly protein 1 like 1, transcript variant X1
XM_023243442.1	−11.97	Lysine demethylase 6B, transcript variant X1	XM_003990793.4	−11.35	Tetratricopeptide repeat, ankyrin repeat and coiled-coil containing 1, transcript variant X2
XM_023243080.1	−11.86	F-box and WD repeat domain containing 5, transcript variant X1	XM_003999386.5	−11.34	ATPase plasma membrane Ca^2+^ transporting 4, transcript variant X2
XM_019826771.2	−11.76	Chromosome A3 C20orf194 homolog, transcript variant X4*	XM_019820383.2	−11.32	Rap associating with DIL domain, transcript variant X14
XM_011288264.3	−11.75	Heterogeneous nuclear ribonucleoprotein K, transcript variant X8*	XM_019826771.2	−11.21	Chromosome A3 C20orf194 homolog, transcript variant X4*

Genes downregulated at both time points are denoted by *.

Among the top 10 genes upregulated at both time points, four were the same, CD63, protein tyrosine phosphatase (transcript variant X18), Colony-stimulating factor 3, and protocadherin gamma C4 (Table 2). While not in the top 10 genes at 2 h, interferon-induced GTP-binding protein Mx1 was the highest induced gene transcript at 17 h.

**TABLE 2:** Top 10 genes upregulated at 2 and 17 h in FCoV infected CRFK cells.

Top 10 Genes Upregulated at 2 h			Top 10 Genes Upregulated at 17 h		
	
*RefSeqID*	*Log fold*	*Description*	*RefSeqID*	*Log fold*	*Description*
XM_019833593.2*	14.42	Protocadherin gamma-C4, transcript variant X28	XM_023238714.1	15.17	Interferon-induced GTP-binding protein Mx1, transcript variant X2
XM_023247173.1	14.30	Rho/Rac guanine nucleotide exchange factor 2, transcript variant X3	NM_001009855.1*	13.94	CD63 molecule
XM_023250856.1*	14.07	Protein tyrosine phosphatase, receptor type S, transcript variant X18	XM_023250856.1*	13.82	Protein tyrosine phosphatase, receptor type S, transcript variant X18
XM_019835257.2	13.27	Neuron navigator 3, transcript variant X1	XM_019833593.2*	13.52	Protocadherin gamma-C4, transcript variant X28
NM_001009227.1*	12.91	Colony stimulating factor 3	XM_003996752.5	13.48	Nuclear factor, erythroid 2 like 1, transcript variant X1
XM_023257557.1	12.78	Alpha 1,4-galactosyltransferase (P blood group), transcript variant X7	XM_023260216.1	13.04	Poly(ADP-ribose) polymerase family member 15, transcript variant X1
NM_001009855.1*	12.74	CD63 molecule	XM_011290734.3	12.70	Epithelial stromal interaction 1, transcript variant X1
XM_019840045.1	12.66	Eukaryotic translation initiation factor 4 gamma 1, transcript variant X4	XM_023250846.1	12.19	Protein tyrosine phosphatase, receptor type S, transcript variant X9
XM_003992443.5	12.63	Thy-1 cell surface antigen, transcript variant X1	NM_001009227.1*	12.09	Colony stimulating factor 3
XM_023238956.1	12.62	Anoctamin 1, transcript variant X7	XM_019817833.1	11.80	Cell division cycle 6, transcript variant X4

Genes upregulated at both time points are denoted by *.

### Macrophage Transcriptomes Show Robust Gene Expression at 2 h in Response to Feline Coronavirus

The transcriptome of macrophages infected with FIPV showed rather individual host responses with few common genes (120 + 100) that were differentially expressed in all of the macrophage samples analyzed (Drechsler et al., 2020). In the current study, we analyzed all of the significantly differentially expressed genes for macrophage samples, to obtain a more complete picture of their responses to coronavirus. At 2 and 17 h respectively, 1313 and 1280 transcripts were downregulated in macrophages infected with FIPV compared to uninfected controls, while 1392 and 1429 transcripts were upregulated (Supplementary Table S8 and Supplementary Figure S2B).

**FIGURE 5:**
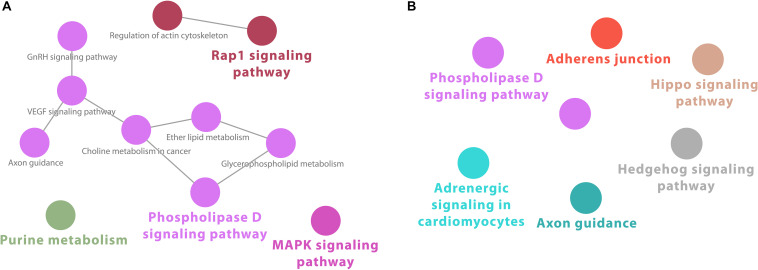
Cytoscape network analysis of **(A)** downregulated and **(B)** upregulated KEGG pathways on infected macrophages at 2 h (adj. *p*-value < 0.05). **(A)** The analysis shows some nodes of the interaction of pathways in the downregulated genes at 2 h. Mainly metabolic pathways, such as “phospholipase D signaling,” “lipid metabolism,” and “choline metabolism in cancer” are intersecting with VEGF and GnRH signaling pathways. **(B)** No network connections were identified for upregulated genes. A multi-color node means that genes within that labeled category are also placed/present in other associated higher hierarchy terms, represented by their respective colors elsewhere in the network.

At 2 h, downregulated genes were enriched for 11 KEGG pathways, whereas for the upregulated ones, the enrichment analysis retrieved 7 pathways (Supplementary Table S9). This is reflected in the network analysis as well, which did show some nodes of the interaction of pathways in the downregulated genes at 2 h (Figure 5A). Mainly metabolic pathways, such as “phospholipase D signaling,” “lipid metabolism,” and “choline metabolism in cancer” are intersecting with VEGF and GnRH signaling pathways. No network connections were identified for upregulated genes (Figure 5B).

**FIGURE 6:**
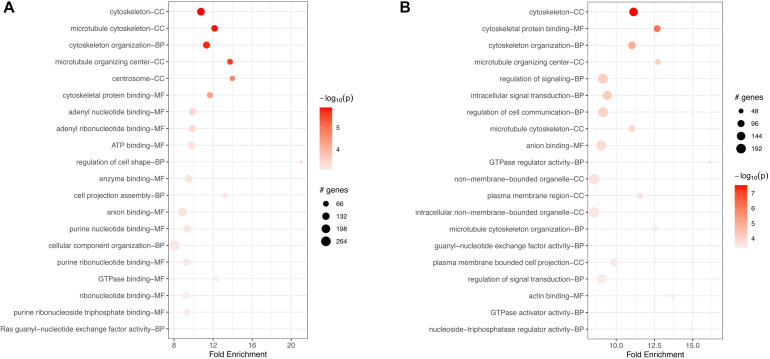
Top 20 most significant GO terms for **(A)** downregulated and **(B)** upregulated genes in FCoV-infected CRFK cells 2 h post-infection (adj. *p*-value < 0.05). In both cases, down- and upregulated genes, the top 20 most significant terms included several cytoskeletal connected functions. **(A)** Downregulated terms were associated mostly with binding of ATP, several nucleotides, and other molecules. **(B)** Upregulated enriched terms included intracellular signal transduction, regulation of cell communication, and signal transduction.

In macrophages, GO-based analyses for both down- and up-regulated genes showed enrichment for hundreds of BP, MF, and CC terms (Supplementary Tables S10, S11). The top 20 terms for each category are shown in Figure 6. In both cases, down- and upregulated genes, the top 20 most significant terms included several cytoskeletal connected functions. Downregulated terms were associated mostly with binding of ATP, several nucleotides, and other molecules (Figure 6A). Upregulated enriched terms included “intracellular signal transduction,” “regulation of cell communication,” and “signal transduction” (Figure 6B).

### Macrophage Transcriptomes Show Downregulation of Immune Pathways and Limited Enrichment of Upregulated Pathways at 17 h Post-infection

**FIGURE 7:**
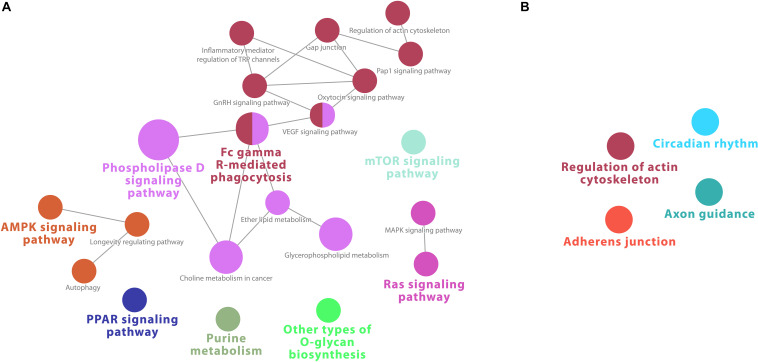
Cytoscape network analysis of **(A)** downregulated and **(B)** upregulated KEGG pathways from infected macrophages at 17 h (adj. *p*-value < 0.05). **(A)** The network analysis shows strong intersecting pathways for downregulated genes, connecting “Fc gamma R-mediated phagocytosis” and several immune signaling pathways with the Phospholipase D pathway and other metabolic pathways. **(B)** No network was present. A multi-color node means that genes within that labeled category are also placed/present in other associated higher hierarchy terms, represented by their respective colors elsewhere in the network.

At 17 h, macrophage downregulated genes were enriched for 23 KEGG pathways, including several immune signaling pathways, such as “PPAR signaling,” “MAPK signaling,” Ras and VEGF signaling pathways, “Fc gamma R-mediated phagocytosis.” Other downregulated pathways were “autophagy” and several metabolic pathways (Supplementary Table S12). Accordingly, the network analysis shows strong intersecting pathways for downregulated genes, connecting “Fc gamma R-mediated phagocytosis” and several immune signaling pathways with the Phospholipase D pathway and other metabolic pathways (Figure 7A).

In contrast to the robust enrichment for downregulated genes, at 17 h, only 4 significant KEGG pathways were retrieved from upregulated genes in macrophages (Supplementary Table S10). No intersecting nodes were found in the network analysis (Figure 7B). “Axon guidance” was again a pathway enriched, in addition to “cytoskeleton,” “adherens junction,” and “circadian rhythm” pathways, but contrary to expectations, no strong immune or virally associated signaling occurred.

**FIGURE 8:**
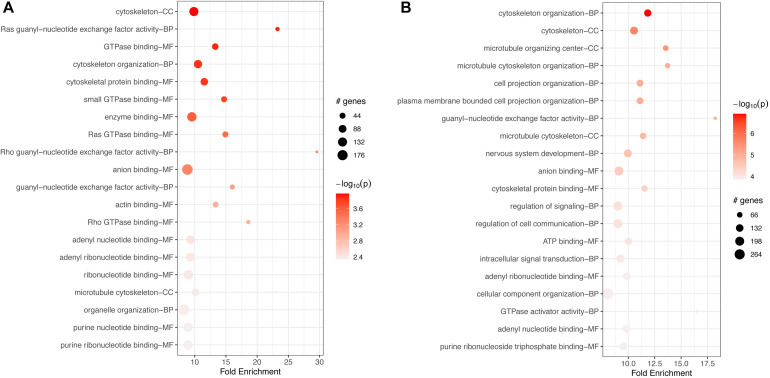
Top 20 most significant GO terms for **(A)** downregulated and **(B)** upregulated genes in FCoV infected macrophages 17 h post-infection (adj. *p*-value < 0.05). **(A)** Downregulated terms were associated mostly with binding of GTPase, nucleotides, and other molecules, such as anion, GTPases. **(B)** Terms enriched for upregulated genes included intracellular signal transduction, regulation of cell communication and signal transduction, and binding of ATP, nucleotides, and other molecules **(B)**.

GO-based enrichment on downregulated genes also showed a long list of over 200 terms for BPs, MFs, and CCs (Supplementary Tables S13, S14) at 17 h. Less than 100 terms were enriched for upregulated genes at 17 h, correlating with the limited KEGG pathway enrichment. The top 20 terms for each category are shown in Figure 8. In both cases, down- and upregulated genes, the top 20 significant terms included several cytoskeletal connected functions, similar to enrichment at 2 h. Downregulated terms were associated mostly with binding of GTPase, nucleotides, and other molecules, such as anion, GTPases (Figure 8A). Terms enriched for upregulated genes included “intracellular signal transduction,” “regulation of cell communication and signal transduction,” and the binding of ATP, nucleotides, and other molecules (Figure 8B).

### Top 10 Genes Down- and Upregulated at Both 2 and 17 h in Macrophages

Among the top 10 downregulated genes, only two were common for both time points, ring finger protein 168, and phosphatidylinositol transfer protein cytoplasmic 1. At both time points, different types of heterogeneous nuclear ribonucleoproteins were also downregulated (Table 3). Significantly upregulated genes that were in the top 10 genes for both timepoints were GRB10 interacting GYF protein 2, CDV3 homolog, and Rap associating with DIL domain (Table 4).

**TABLE 3:** Top 10 genes downregulated at 2 and 17 h in FCoV infected macrophages.

Top 10 Genes Downregulated at 2 h			Top 10 Genes Downregulated at 17 h		
	
*RefSeqID*	*Log fold*	*Description*	*RefSeqID*	*Log fold*	*Description*
XR_002739150.1	−9.64	Heterogeneous nuclear ribonucleoprotein U, transcript variant X2	XM_003985246.5	−12.18	Heterogeneous nuclear ribonucleoprotein D, transcript variant X1
XM_019839937.2*	−9.54	Ring finger protein 168, transcript variant X2	XM_019819663.2	−10.47	URI1, prefoldin like chaperone, transcript variant X2
XM_019818127.2	−9.33	Phosphatidylinositol transfer protein, cytoplasmic 1, transcript variant X2	XM_019836465.2	−10.12	FGR proto-oncogene, Src family tyrosine kinase, transcript variant X7
XM_023243225.1	−9.26	Semaphorin 4D, transcript variant X4	XM_019818127.2*	−10.03	Phosphatidylinositol transfer protein, cytoplasmic 1, transcript variant X2
XM_011289199.3	−8.96	Sperm associated antigen 9, transcript variant X11	XM_023252877.1	−10.02	3′-phosphoadenosine 5′-phosphosulfate synthase 1, transcript variant X3
XM_023238621.1	−8.94	Solute carrier family 19 member 1, transcript variant X2	XR_002737850.1	−9.79	Uncharacterized LOC111557626, transcript variant X1
XR_002742425.1	−8.82	Centrosomal protein 85 like, transcript variant X2	XM_023260369.1	−9.71	ATPase phospholipid transporting 11B (putative), transcript variant X6
XM_019818372.2	−8.78	Regulatory associated protein of MTOR complex 1, transcript variant X3	XM_019830307.1	−9.63	Pre-mRNA processing factor 4B, transcript variant X1
XM_006940110.4	−8.72	Synergin gamma, transcript variant X13	XM_023245511.1	−9.56	Ankyrin repeat domain 11, transcript variant X3
XM_023257518.1	−8.7	*X*-prolyl aminopeptidase 3, transcript variant X4	XM_019839937.2*	−9.54	Ring finger protein 168, transcript variant X2

Genes downregulated at both time points are denoted by *.

**TABLE 4:** Top 10 genes upregulated at 2 and 17 h in FCoV infected macrophages.

Top 10 Genes Downregulated at 2 h			Top 10 Genes Downregulated at 17 h		
	
*RefSeqID*	*Log fold*	*Description*	*RefSeqID*	*Log fold*	*Description*
XM_019826402.2	9.26	Zinc finger MYND-type containing 8, transcript variant X6	XM_023252992.1	12.36	Heterogeneous nuclear ribonucleoprotein D, transcript variant X6
XM_019838883.2 *	9.21	GRB10 interacting GYF protein 2, transcript variant X7	XM_023260549.1*	11.93	CDV3 homolog, transcript variant X1
XM_023260549.1*	9.18	CDV3 homolog, transcript variant X1	XM_019838883.2*	10.9	GRB10 interacting GYF protein 2, transcript variant X7
XM_019830298.2	9	ZFP62 zinc finger protein, transcript variant X4	XM_023247167.1	10.81	Immunoglobulin superfamily member 9, transcript variant X2
XM_023251263.1	8.87	RB transcriptional corepressor like 1, transcript variant X3	XM_023245341.1	10.8	CCCTC-binding factor, transcript variant X1
XM_023254208.1	8.67	Crystallin beta-gamma domain containing 1, transcript variant X2	XM_023256742.1_	10.76	Peroxisomal biogenesis factor 5, transcript variant X1
XM_023254423.1	8.64	Semaphorin 6A, transcript variant X3	XM_023244663.1	10.71	Acetyl-CoA carboxylase alpha, transcript variant X8
XM_011281322.1	8.58	Lipin 1, transcript variant X3	XM_023242979.1	9.95	Vav guanine nucleotide exchange factor 2, transcript variant X7
XM_006941891.3*	8.54	Rap associating with DIL domain, transcript variant X11	XM_011283119.3	9.85	Chromodomain helicase DNA binding protein 8, transcript variant X1
XM_003990727.5	8.52	Cyclin T2, transcript variant X2	XM_006941891.3*	9.81	Rap associating with DIL domain, transcript variant X11

Genes downregulated at both time points are denoted by *.

**FIGURE 9:**
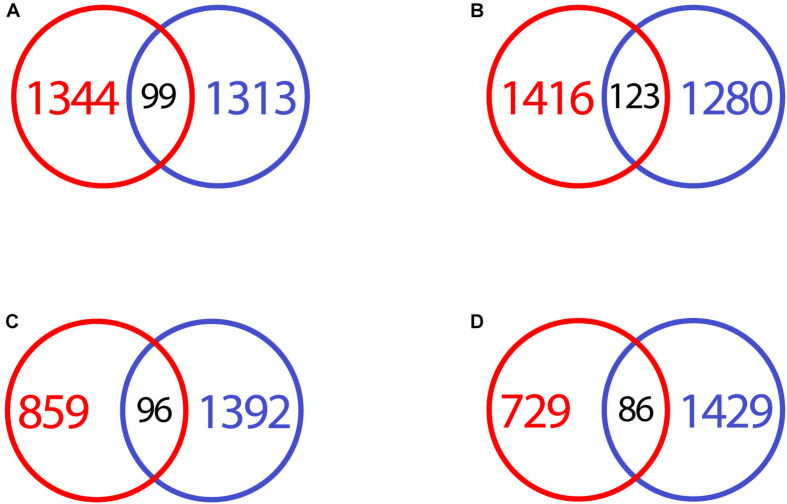
VennDiagram of differentially expressed genes in infected CRFK and macrophages, compared at different time points. **(A)** Downregulated genes CRFK (red) vs. macrophages (blue) at 2 h after infection. **(B)** Upregulated genes CRFK (red) vs. macrophages (blue) at 2 h after infection. **(C)** Downregulated genes CRFK (red) vs. macrophages (blue) at 17 h after infection. **(D)** Upregulated genes CRFK (red) vs. macrophages (blue) at 17 h after infection.

CRFK and macrophage differentially expressed genes were very different in response to feline coronavirus. When transcripts were compared at each time point, there was very little overlap. While several hundred to over a thousand transcripts were down- and upregulated at both time points in both cell types, the overlap of genes was minimal (Figure 9). Within the intersecting genes, only the KEGG pathway “bile secretion” was enriched at 17 h for downregulated transcripts, with just 3 common genes found.

## Discussion

The pathogenesis of FIPV strains is still not well understood, but a predominant theory has been that the pathogenic FIPV had mutated from the benign intestinal FECV such that it could acquire tropism for monocytes/macrophages. The implication was that FECV was present only in the intestines, while FIPV would now replicate mainly in monocytes, and that the infected monocytes subsequently cause the disease by transporting the virus systemically. However, FECV has been shown to be present systemically in monocytes (Kipar et al., 2010; Vogel et al., 2010) and FIPV can be present in the intestines without being infectious (Pedersen et al., 2012). *Ex vivo* studies on monocytes/macrophages host responses have been limited by the fact that the presence of the virus is extremely low, in only about 0.2–2% of infected cells (Dewerchin et al., 2005). We previously demonstrated that at 2 h, the virus is taken up by the macrophages, as evidenced by the presence of viral RNA, but were not able to observe a significant increase of viral RNA at 17 h using RNA sequencing, while even limited uptake of the virus into CRFK cells led to several log fold of replication (Drechsler et al., 2020). This discrepancy in viral amplification in the two cell types, combined with their different function, most likely accounts for the pronounced difference observed in gene expression in this analysis.

When comparing host responses in CRFK to primary macrophages, many differences were observed with regards to expressed genes and their enrichments for both KEGG pathways and GO terms. Comparing downregulated gene pathways and ontologies, there were fewer significant pathways found in the CRFK cells than in macrophages. The two enriched downregulated pathways in CRFK 2 h post-infection observed were “axon guidance,” and “choline metabolism in cancer,” and were also downregulated in macrophages. In macrophages, those two pathways were part of a larger network of connected pathways. At 17 h, this difference was even more pronounced, with few pathways enriched for CRFK, while macrophage pathways form a larger network of several pathways. The only common pathway enriched for the two cell types at this time point was “MAPK signaling,” which is also connected to the KEGG pathway “axon guidance,” as MAP kinases play an important role in neuron growth and regeneration (Raivich and Makwana, 2007).

Comparing the 20 most significant gene ontology terms, CRFK terms were mostly found in the categories of neuronal development, differentiation, and cellular organization at both timepoints. For macrophages, similar terms were found mostly in regards to cytoskeletal function. Different terms included nucleotide and other molecule binding functions, and less enrichment for neuronal differentiation and development were found in the macrophages.

It has been shown that viral miRNAs of DNA viruses, such as herpesvirus, interfere with several host signaling pathways, including axon guidance, cell growth, and cell differentiation (Carl et al., 2013). Herpesviruses are not necessarily replicating in neurons though, but rather enter and go latent, so interfering with neuronal pathways might be a feature of latency in those cases. Recent studies imply that RNA viruses potentially also generate these viral miRNAs (Rouha et al., 2010) and it would appear that these consequently try to interfere with the host signaling. Up to one-third of cats with FIP manifest with neurological signs (Foley et al., 1998) and coronaviruses of several species have been shown to be neurotropic (Bergmann et al., 2006). Therefore, it is of interest that this pathway is targeted by the virus. Further studies are needed to better understand the viral host interaction with regards to these neuronal pathways, particularly considering that this was more pronounced in CRFK, and less so in macrophages. Another function that was clearly targeted was cytoskeleton and associated functions. Viruses have evolved mechanisms to counter the host response, including the reorganization of host organelles to form viral replication sites, generation of viral countermeasures, and the assembly, transport, and release of new viral particles (Naghavi and Walsh, 2017). This involves the host’s cytoskeleton and microtubules, which is what was seen in the gene expression in response to the virus in both cell types. By differentially regulating these pathways, the virus is hijacking the normal functions involved in cellular homeostasis and using them to its advantage.

Among the 10 most significantly downregulated genes at 2 or 17 h, there was no overlap between the cell types. In both, CRFK and macrophages, members of the heterogeneous nuclear ribonucleoprotein family were downregulated. However, the subtypes were different, hnRNP K in CRFK and hnRNP U and D respectively in macrophages. Proteins from the hnRNP family play important roles in mRNA processing, RNA metabolism, and transport and are host factors important in the replication of RNA viruses (Dinh et al., 2013; Chang et al., 2017), so it does not seem surprising that the transcription of several of these proteins appears to be differentially regulated in both cell types by FIPV. Further investigation of specific subtypes and their role in feline coronavirus is warranted. In CRFK, the top downregulated gene at 17 h was talin 1, an integrin-associated protein needed for all integrin adhesive functions (Klapholz and Brown, 2017). Integrins play an important role in cell signaling and recent research indicates that Hepatitis B degrades talin 1 to facilitate its replication (van de Klundert et al., 2016), but much is still unknown about this protein and viral infection. In summary, many more genes and pathways are strongly downregulated in macrophages, but not CRFK, and there is almost no commonality in regards to those genes and pathways.

Comparing CRFK and primary macrophage upregulated genes and enriched pathways following infection, the picture is reversed. For both time points, a large number of pathways was enriched both at 2 and 17 h for CRFK, leading to the integration of several pathways into networks, which is particularly pronounced at 17 h in the cell line. In contrast, few pathways were enriched for upregulated genes in macrophages at either timepoints and no networks were generated in the analysis. When KEGG pathway enrichment was compared across both time points, only axon guidance appeared at both time points for both cell types. None of the many viral pathways associated with FCoV replication in CRFK were upregulated in macrophages. While strong induction of immune and viral pathways was seen at the 17 h time point in CRFK, in macrophages even fewer pathways were enriched at this timepoint than compared to 2 h.

Comparing the most significant gene ontology, terms again were mostly found in the categories of neuronal development, differentiation, and cellular organization at both time points in CRFK. Additionally, “intracellular signal transduction” and at 17 h “response to virus,” “defense response to virus,” and “semaphoring-plexin signaling pathway” were found. For macrophages, terms were found mostly in regards to cytoskeletal function, and at 2 h, also several terms related to intracellular signal transduction. Different terms in macrophages included nucleotide and other molecule binding functions, and no enrichment for a “response to virus” or neuronal differentiation and development were observed in the macrophages.

Among the 10 most significantly upregulated genes at 2 or 17 h, there was no overlap between the cell types. The most significantly upregulated gene in CRFK at 17 h, is interferon-induced GTP-binding protein Mx1 which is associated with viral replication and the antiviral host response (Pillai et al., 2016). In macrophages, the most significant gene upregulated at this time point is hnRNP D, which as previously stated, is part of the RNA binding protein family involved in RNA processing. In summary, the response of CRFK in response to viral infection is quite expected, with strong upregulation of both viral- and immune-related KEGG pathways and GO biological processes. In contrast, the response in macrophages is not typical of a cell infected with virus, despite uptake of virus evidenced by viral reads in the cell, and needs further investigation.

Crandell-Rees Feline Kidney cells, derived from the feline kidney cortex with epithelial morphology, are permissive for feline coronavirus replication (Stoddart and Scott, 1989; Dewerchin et al., 2005). Macrophages are innate immune cells generally hard to infect *in vitro* (Stoddart and Scott, 1989; Dewerchin et al., 2005; Shirato et al., 2018). While epithelial cells generally function as part of the innate immune system in a wider sense, only macrophages are professional antigen-presenting cells and considered one of the most critical players in host–pathogen interactions. Epithelial cells, in general, are targets of coronavirus infection and CRFK cells have been used as the primary way to grow the virus in cell culture. However, it appears that macrophages might not serve as a primary cell to replicate the virus, and rather play a different role in the pathogenesis of the disease. This would have to be further investigated *in vivo*, where monocytes have been shown to be viremic, and release inflammatory factors that cause vasculitis, a key clinical factor in disease (Dewerchin et al., 2005). It is conceivable that macrophages serve as a vehicle to transport the virus and consequently interact with or activate T cells. T cells are depleted in FIP affected cats, but the underlying mechanism is also not well understood. It has been suggested that macrophages are responsible for the apoptosis of T cells (Haagmans et al., 1996), so further investigations of macrophage gene expression and the effect on T cells will be needed. Gene expression of monocytes/macrophages can give us further insight into the early responses of these critical immune cells to infection and subsequently elucidate the pathogenesis of FCoV infection leading to T cell depletion, inflammatory responses, and clinical disease. Comparisons of gene transcription in these immune cells important for antigen presentation and interaction with other immune cells to epithelial cells which are permissive for productive replication can possibly lead to discerning pathways relevant to pathogenesis in the early stages of infection.

## Data Availability Statement

The raw datasets presented in this study are publicly available through NCBI-SRA repository under the PRJNA668739 BioProject accession number.

## Ethics Statement

The animal study was reviewed and approved by the Institutional Animal Care University Committee (IACUC) of Western University of Health Sciences, protocol approval number R10/IACUC/017.

## Author Contributions

YD and PD: conceptualization and funding acquisition. YD and LG: methodology and validation. EV: software and data curation. EV and YD: formal analysis and visualization. LG and YD: investigation. YD: writing—original draft preparation, supervision, and project administration. YD, EV, LG, and PD: writing—review and editing. All authors: read and agreed to the published version of the manuscript.

## Conflict of Interest

The authors declare that the research was conducted in the absence of any commercial or financial relationships that could be construed as a potential conflict of interest.

## Footnotes

1 www.bioinformatics.babraham.ac.uk/projects/fastqc

2 http://geneontology.org/

3 https://www.genome.jp/kegg/pathway.html

## Supplementary Material

Supplementary Figure 1 Multidimensional scaling (MDS) analysis on RNA-seq samples from feline CRFK cells infected with feline infectious peritonitis virus (FIPV) at two different time points (2 and 17 h). Colors indicate infection status: Non-infected (green), 2 h of infection (blue), and 17 h of infection (red).

Supplementary Figure 2 Gene expression profile of all DEGs individually represented across all samples assessed for CRFK cells **(A)** and Macrophages **(B)**. Transcripts per million (TPM) normalized expression values were used on a log10 scale, being TPMs less than 1 represented by bluish shading, whereas equal to or greater than 1 by pink/reddish shading. Hierarchical clustering was performed on both columns (samples) and rows (transcripts), being represented by dendrograms on the top and left-hand side of each panel, respectively. Control, 2 h of infection, and 17 h of infection sample groups are labeled with “Ctrl,” “T2,” and “T17,” respectively.
