# Supplementary figures and images for: CRFK and Primary Macrophages Transcriptomes in Response to Feline Coronavirus Infection Differ Significantly
Source: Front Genet. 2020 Dec 3;11:584744. doi: 10.3389/fgene.2020.584744 (PMC7745755; doi:10.3389/fgene.2020.584744)

CRFK MDS plot

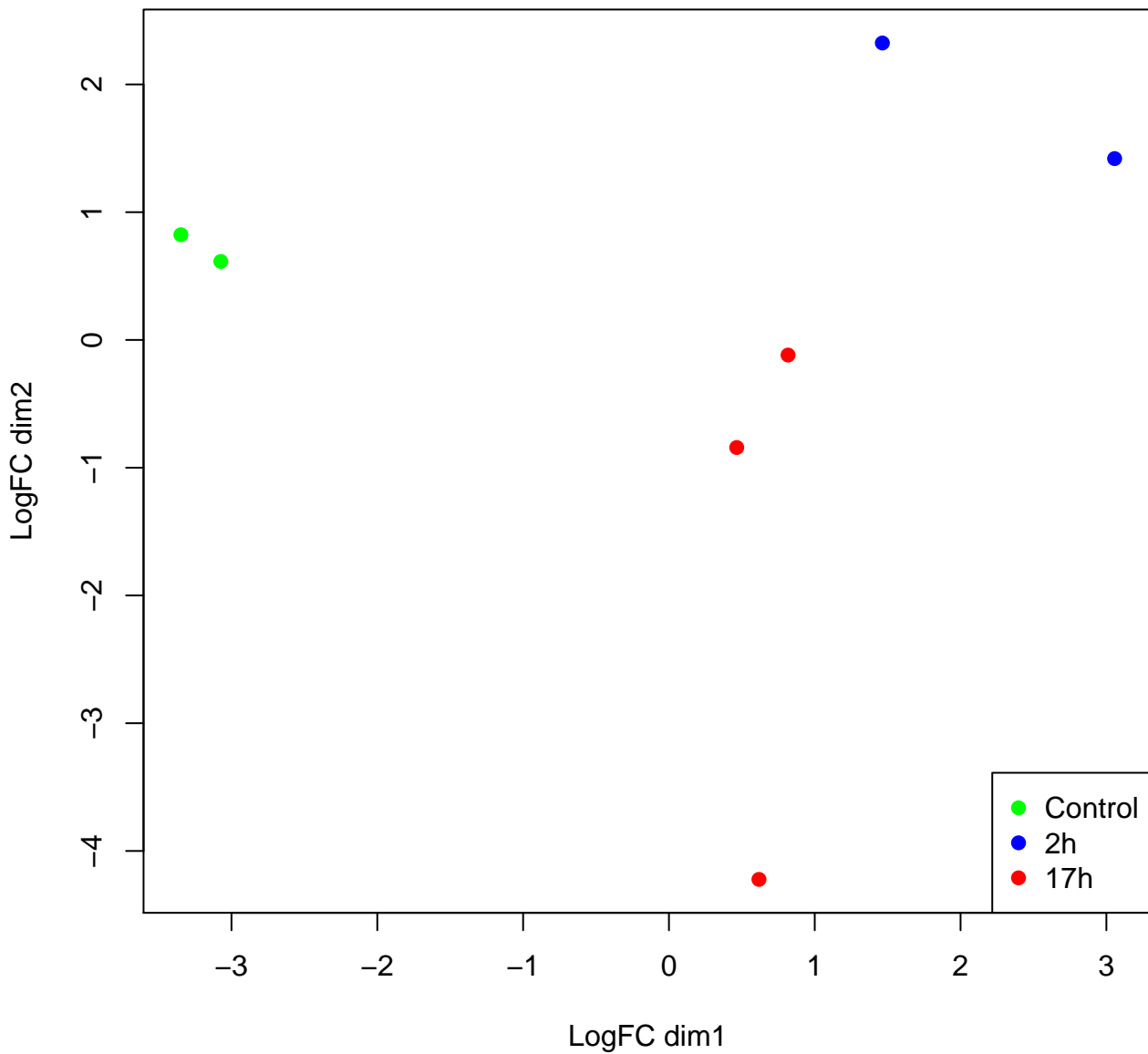

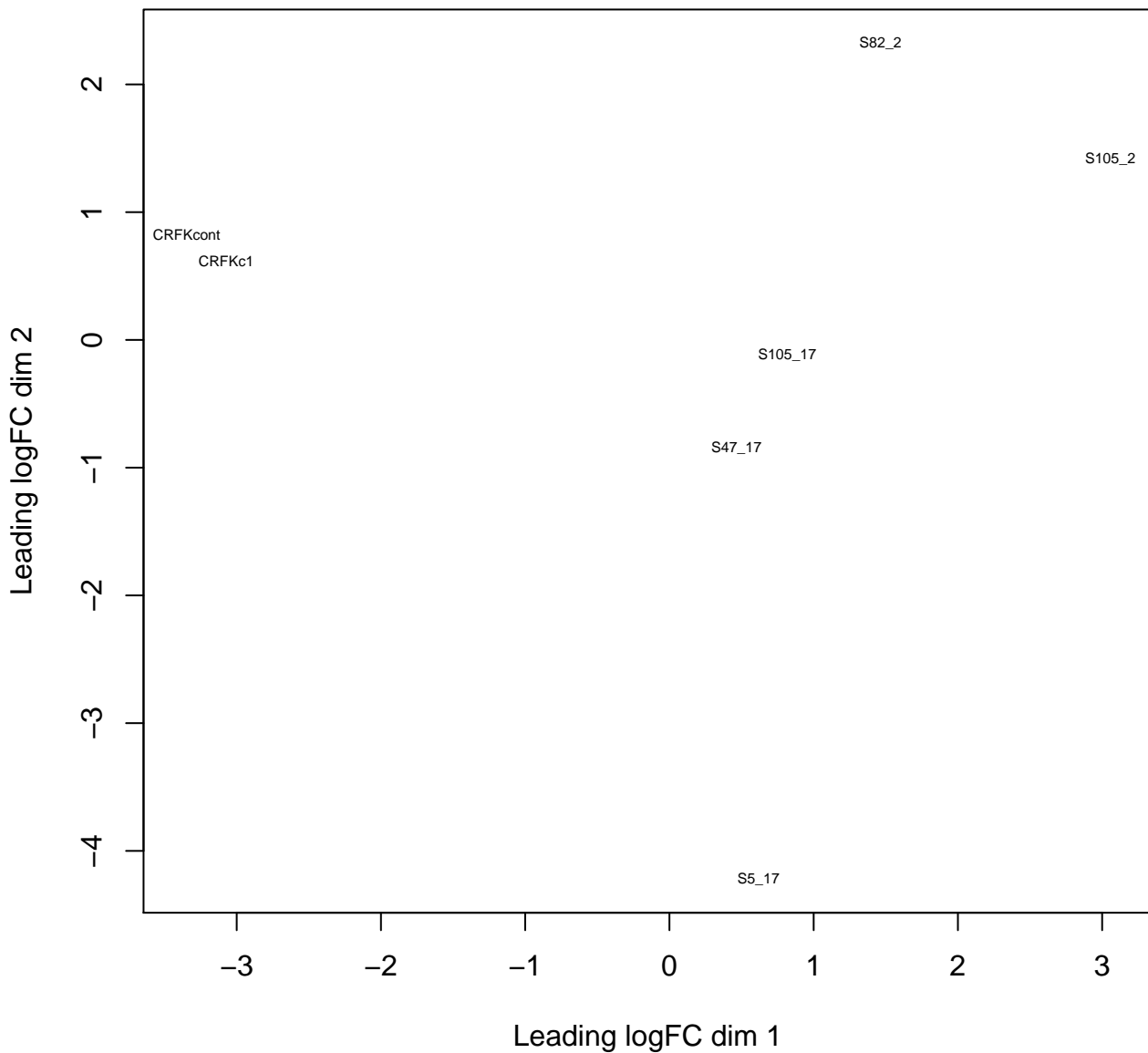

Supplement: Supplementary Figure 1 — Multidimensional scaling (MDS) analysis on RNA-seq samples from feline CRFK cells infected with feline infectious peritonitis virus (FIPV) at two different time points (2 and 17 h). Colors indicate infection status: Non-infected (green), 2 h of infection (blue), and 17 h of infection (red). [file Presentation_1.zip › Suppl. Figure 1.PDF]

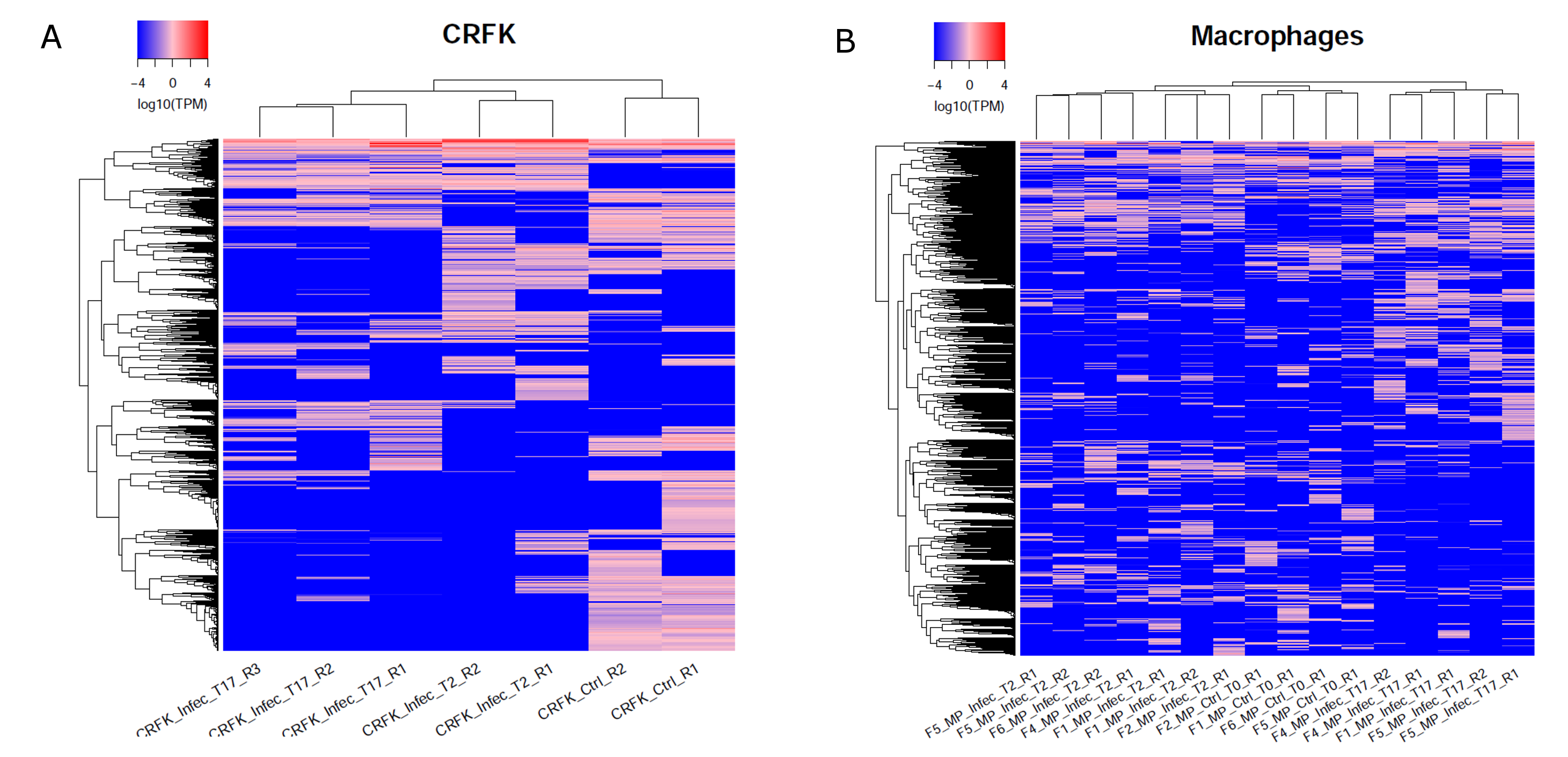

Supplement: Supplementary Figure 1 — Multidimensional scaling (MDS) analysis on RNA-seq samples from feline CRFK cells infected with feline infectious peritonitis virus (FIPV) at two different time points (2 and 17 h). Colors indicate infection status: Non-infected (green), 2 h of infection (blue), and 17 h of infection (red). [file Presentation_1.zip › Suppl. Figure 2.TIFF]
